# A Novel Method for the Synthesis of ^99m^Tc-Ofloxacin Kits Using D-Penicillamine as Coligand and Their Application as Infection Imaging Agent

**DOI:** 10.1155/2015/502680

**Published:** 2015-05-19

**Authors:** Muhammad Abdul Qadir, Shabnam Shahzad, Rashid Rasheed, Mahmood Ahmed, Shahzad Anwar, Syeda Kiran Shahzadi

**Affiliations:** ^1^Institute of Chemistry, University of the Punjab, Lahore 54590, Pakistan; ^2^Gujranwala Institute of Nuclear Medicine and Radiotherapy, Gujranwala, Pakistan

## Abstract

The employment of radiopharmaceuticals is increasing nowadays for infection imaging and early execution of patients having infectious or inflammatory complaints. The main aim of this study was to discover a novel method for the labeling of ofloxacin with ^99m^Tc, optimization of labelling conditions to get higher percent yield, to assess kits radiochemical purity, in vitro stability, partition coefficient, protein binding, and intracellular accumulation in *Pseudomonas aeruginosa*, *Salmonella typhi*, and *Escherichia coli* in infected rabbits. Maximum labeling efficiency was achieved when 1.5 mg ofloxacin was labeled with 10–20 mCi sodium pertechnetate in the presence of 3 mg D-penicillamine, 75 *μ*g SnCl_2_. In vitro binding and biodistribution in *Pseudomonas aeruginosa, Salmonella typhi,* and *Escherichia coli* showed good results. This new complex is efficient for the imaging of infections caused by Gram-positive and Gram-negative bacteria.

## 1. Introduction 

Radiopharmaceuticals should possess certain properties such as high percent labeling, fast accumulation at infection site, low toxicity, rapid blood clearance, high target to nontarget ratios, and low cost [1]. Many imaging techniques for diagnosis of infection such as computer tomography (CT), magnetic resonance imaging (MRI), ultrasonography (US), and X-rays are commercially available and used for the detection of infection. But these tools are not appropriate for the diagnosis and localization of infection at early stages because they cannot reveal the changes in the morphology of tissues due to abscess formation [2–4]. Different radiopharmaceuticals were developed and used for infection imaging such as ^67^Ga-citrate and ^111^In-labeled leukocytes but limitations and inconvenience related to them, that is, long gamma rays exposure, nonavailability of their generators, risk of contamination, and nonspecificity, make it a great challenge to select and synthesize a proper radiopharmaceutical [5–8]. Among all the conventional radionuclides available in the market ^99m^Tc is most favorable due to its short half-life and availability [9–13].

The radiolabeled antibiotic kits will be considered ideal if it is easily metabolized, has rapid clearance out of body, and has high level of localization at infection site. Certain quinolones that have broad spectrum such as norfloxacin, sitafloxacin, ciprofloxacin, enrofloxacin, ceftriaflaxone, lemofloxacin, difloxacin, pefloxacin, and ceftizoxime are extensively used in the treatment of serious bacterial infection [14–18].

^99m^Tc-ciprofloxacin is an important commercially available radiopharmaceutical that is used as infection imaging agent [19–22]. On the other hand in vivo study of ciprofloxacin showed contradictions in infection specificity [23–27] and drawbacks in the procedure of its kit development [10, 27–29].

**Figure 1:**
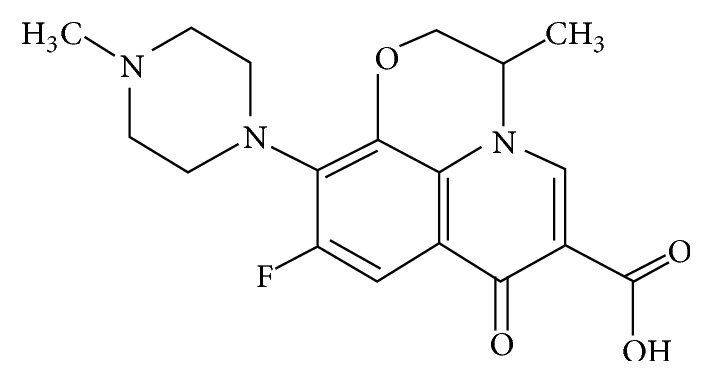
Structure of ofloxacin.

Ofloxacin is very potent member of fluoroquinolone family with broad spectrum against a wide variety of bacterial infections; its structure is shown in Figure 1. Literature reveals that there are problems in its radiolabeling with ^99m^Tc such as use of HCl, heating, and instability in serum; thus there is a need to search more efficient, robust method for the development of ofloxacin kits, its radiolabeling, and in vivo studies to sketch the whole profile of the pharmacokinetics and efficacy. Therefore the purpose of the presented work was to develop a simple and well-organized method at room temperature and pressure (without HCl, heating) to develop ^99m^Tc-ofloxacin, its optimization producing high labeling efficiency and greater stability (low colloidal in serum), and its biodistribution in* Salmonella typhi*,* Pseudomonas aeruginosa*, and* E. coli* infected rabbits.

## 2. Materials and Methods

D-Mannitol (Sigma Aldrich, USA), sodium pyrophosphate (RDH, Germany), octanol (Sigma Aldrich, USA), acetone (Merck, Germany), D-penicillamine (Sigma Aldrich, USA), stannous chloride (Sigma Aldrich, USA), gentistic acid (Sigma Aldrich, USA), ascorbic acid (MP Biomedical Inc.), trichloroacetic acid (Fisher scientific, UK), saline (Otsuka, Pakistan), ofloxacin (donated by Java pharmaceutical, Lahore, Pakistan), and 0.22 *μ*m filter (MS Nylon Membrane Filters, USA) were used in present study.

### 2.1. Formulation of Ofloxacin Kits

Optimization studies were performed to find out the best conditions for the labeling of ofloxacin with technetium. For this purpose SnCl_2_, amount of antibiotic, pH, and reaction time were studied.

#### 2.1.1. Effect of Reducing Agent

Amount of reducing agent was varied between 25 and 300 *μ*g. It was found that maximum labeling efficiency was obtained at 75 *μ*g; below and above this labeling efficiency was low. All the tests were performed three times (*n* = 3). Results are summarized in Table 1.

#### 2.1.2. Effect of Ofloxacin Amount

Labeling efficiency was analyzed at 0.5–2.5 mg of ofloxacin. It was found that, at 1.5 mg ofloxacin, maximum labeling efficiency was obtained. Results are given in Table 1.

#### 2.1.3. Effect of pH on Labeling Efficiency

**Figure 2:**
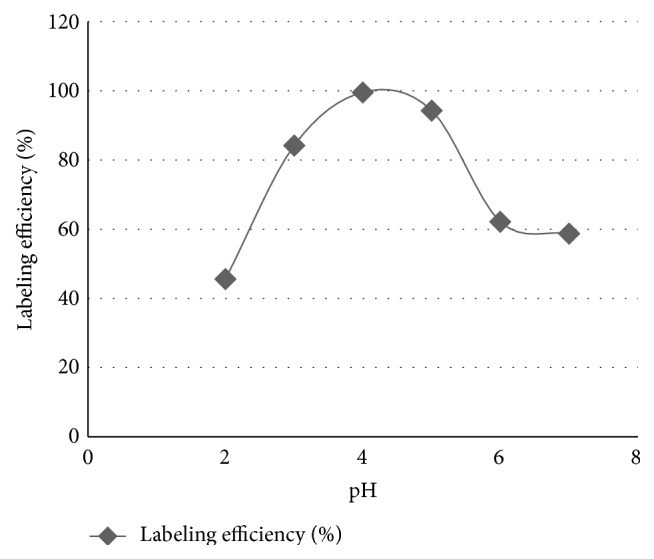
pH Effect on labelling efficiency of ^99m^Tc-ofloxacin.

Labeling efficiency (LE) was tested between 2, 3, 4, 5, 6, and 7 pH values and it was concluded that pH 4 was best to get highest value of LE. The results are reported in Figure 2.

#### 2.1.4. Effect of Incubation Time on Labeling Efficiency

Labeling efficiency was checked at 5, 10, 15, 20, 25, and 30 minutes. 20 minutes is the ideal incubation time to get highest labeling efficiency. To formulate the ofloxacin kit 1.5 mg ofloxacin, 2.0 mg gentistic acid, 300 *μ*g D-penicillamine, and 20 mg D-mannitol were dissolved in 1.0 mL distilled water with continuous stirring (Solution A). Then stannous chloride, 75 *μ*g (15 *μ*L) from a solution having 50 mg SnCl_2_, and 50 mg Sod/Pot pyrophosphate in 10 mL distilled water were added dropwise to Solution A (Table 1). The pH was set at 4 by 0.1 N NaOH/0.1 NHCl. This kit solution was filtered by a 0.22 *μ*m filter. Kits were prepared, lyophilized, and stored at 4°C. Kit's development was accomplished under sterile circumstances in a laminar flow cabinet at room temperature.

### 2.2. Radiolabeling with ^99m^Tc

Freshly eluted sodium pertechnetate (370–740 MBq) from in-house iv)packgen ^99m^Tc-generator was added to freeze dried kits by insulin syringe and incubated at room temperature.

### 2.3. Radiochemical Purity Assessments

ITLC, PC, and HPLC were used to assess the radiochemical purity of the ^99m^Tc-ofloxacin. Paper chromatography (PC) was used to calculate free pertechnetate (^99m^TcO_4_^−^); instant thin layer chromatography (ITLC) was used to calculate the hydrolyzed/reduced technetium (^99m^TcO_2_). Counts of ^99m^Tc-ofloxacin were measured through a radioactive detector, that is, Berthold (LB-507) and NaI (Tl) scintillation detector in a Reverse Phase HPLC (RP-HPLC); Perk Elmer (L7200). Nucleosil C-18 column (100 Å, 5 *μ*m, 250 × 4 mm) was used for analytical separations. The solvents variable gradients are 95% A and 5% B at 0 min, 95% A and 5% B at 5 min, 0% A and 100% B at 25 min, and 0% A and 100% B at 30 min with rate of flow 0.5 mL per min. The solvent detail is A = 0.1% tetrafluoroacetic acid in H_2_0 and B = acetonitrile.

### 2.4. In Vitro Stability Studies

Stability studies were performed at room temperature and in fresh human blood serum at 37°C and labeling efficiency was calculated at regular intervals after 1 h, 2 h, 3 h, 4 h, 5 h, and 6 h (Table 2).

### 2.5. Partition Coefficient

Octanol/saline was used as organic and inorganic layers in order to calculate partition coefficient. Counts in 100 *μ*L of both inorganic and organic layers were calculated with well-type (NaI) *γ*-counter. Partition coefficient values are calculated by mean (±SD, *n* = 3).

Consider the following: Log*P* value: Log (% in octanol/% in saline).

### 2.6. Protein Binding

Protein binding of the labeled complex was measured by incubating it with fresh human blood for 1 h, placing it in a preset water bath (37°C) for ten minutes, centrifuging at 3000 rpm, including equal volume of 10% “trichloroacetic acid” (TCA), and again centrifuged. Afterward both supernatant and residue were counted for radioactivity.

### 2.7. In Vitro Binding of the Complex

It was studied against three bacterial strains, that is,* Salmonella typhi*,* Pseudomonas aeruginosa*, and* E. coli*, using cylinder plate method of microbiological assay (used for the potency determination of antibiotics) [30].

### 2.8. Biodistribution in Normal and Infected Rabbits

For normal biodistribution 1.0 mL (2 mCi) of ^99m^Tc-ofloxacin was injected in the iliac vein of rabbit after valium (1.0 mL) anesthesia. Scintigraphic images were taken at 30 min, 1 h, 2 h, 3 h, and 4 h after injection with Xeleris 2.0523 Gamma camera (Figure 4).

For biodistribution study in infected rabbits, 1.0 mL suspension (3 × 10^8^ cfu/mL) of* Salmonella typhi*,* Pseudomonas aeruginosa*, and* E. coli *was injected in three different rabbits (thigh muscles). After the initiation of infection and swelling at the infected site of rabbits, ^99m^Tc-ofloxacin (1.0 mL = 2.5 mCi) was injected. Scintigraphy images were taken at 1 h–4 h and 24 h after injection with Infinia Dual head Gamma Camera, that is, Figures 5, 6, and 7.

## 3. Results and Discussions

Structure of ofloxacin shows the electron donor species, that is, oxygen, nitrogen, and sulphur, and these species can easily react with reduced sodium pertechnetate and a complex is formed [31, 32] (Figure 1).

Optimization of labeling conditions revealed that highest labeling efficiency was achieved when 1.5 mg ofloxacin was labeled with sodium pertechnetate (370–740 MBq) in the presence of 75 *μ*g stannous chloride at pH 4 and incubation time is 20 minutes. The reaction is favorable at acidic pH but shifting towards basic pH, labeling efficiency was decreased (Figure 2).

**Figure 3:**
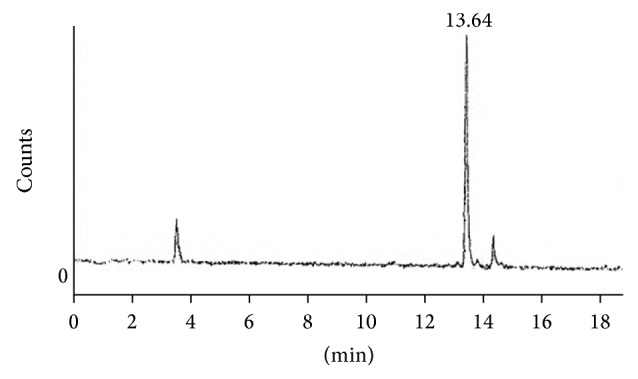
Chromatogram showing a single large peak of ^99m^Tc-ofloxacin while two small peaks showing reduced and free pertechnetate.

HPLC studies of the complex depict that a single compound is formed after this reaction and its retention time was 13.64 min which also matches with the reported value in literature [31] and yield was >96% (Figure 3). In ITLC, saline was used as solvent; very small activity remained at origin corresponding to reduced ^99m^Tc. In PC acetone was used as solvent; small amount of the activity was moved which belonged to free ^99m^TcO_4_.

In vitro stability tests at room temperature and in human serum at 37°C showed consistency for 6 h (Table 2) with no significant disintegration of the complex. It confirmed that colloidal impurity and instability which was issue in earlier studies were removed [24, 33–35]. Low lipophilicity (Log*P* = −2.58) and Log*P* value suggests that dilution of injection will be made with saline.

**Figure 4:**
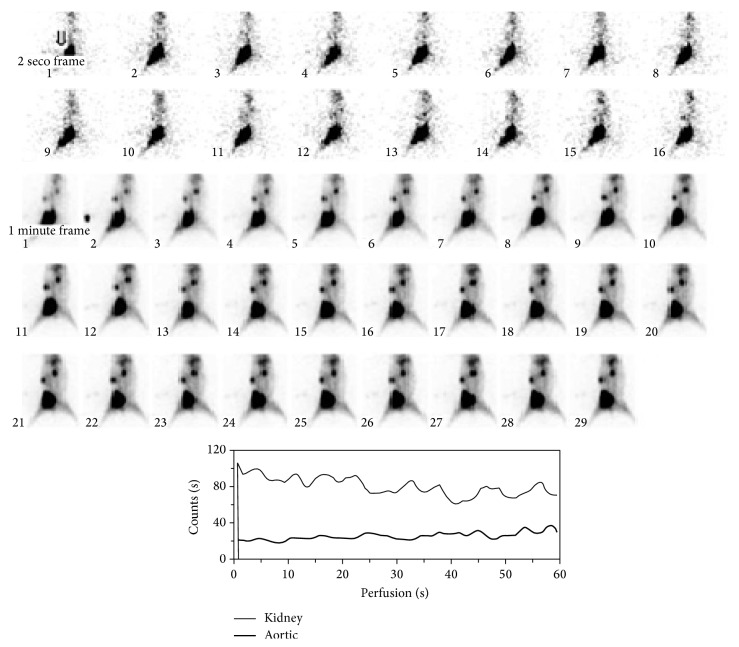
Biodistribution of ^99m^Tc-ofloxacin in normal rabbit (perfusion 0–30 sec.).

**Figure 5:**
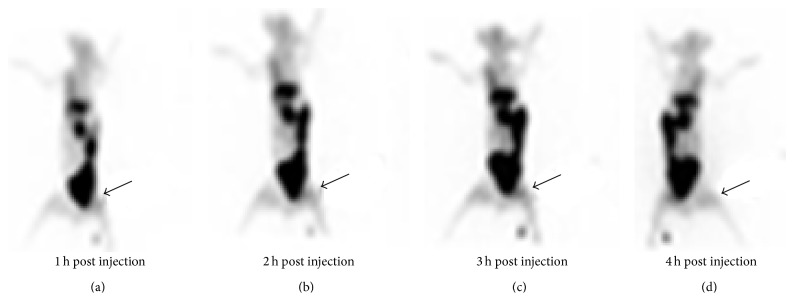
Scintigraphy images of rabbit (infection in right thigh muscle) at 1 hr-2 hrs in* E. coli*.

**Figure 6:**
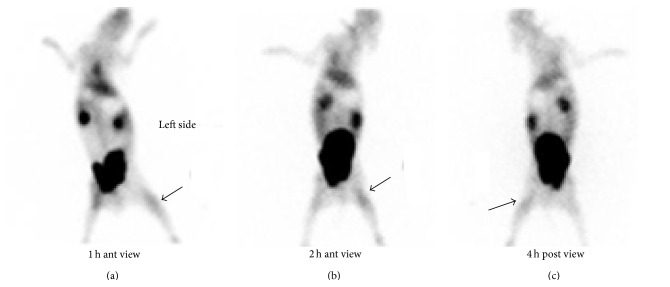
Scintigraphy image of biodistribution of* Pseudomonas aeruginosa* in rabbit (left thigh muscle infection) at 1 hr–4 hrs.

**Figure 7:**
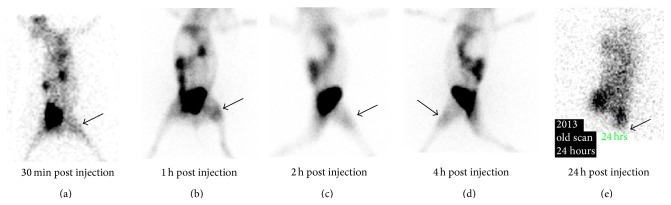
Scintigraphic images of biodistribution of ^99m^Tc-ofloxacin in* Salmonella typhi* at 30 min–24 hrs.

At first hour radioligand was accumulated in the liver and spleen but excreted out rapidly after 4 hr as shown in scintigraphic images (Figure 4). The complex showed moderate binding with serum proteins, that is, 52%, which could explain its rapid washout from body organs. In vitro binding with* Escherichia coli*,* Salmonella typhi*, and* Pseudomonas aeruginosa* showed encouraging results. Biodistribution in normal rabbit showed higher value of activity in kidney and bladder and it is suggested that medicine is excreted mainly through this pathway (Figure 4). The target to nontarget (*T*/*NT*) ratios for* Escherichia coli* were 2.39 ± 0.17 and 1.8 ± 1,* Pseudomonas aeruginosa* 3.63 ± 0.16 and 3.13 ± 0.18, and* Salmonella typhi *4.47 ± 0.19 and 4.06 ± 0.20 (Tables 3, 4, and 5) at 1 h–4 h after injection which shows the presence of significant amount of complex at infection site that indicates its specific binding with these strains of bacteria. It was also observed that activity uptake was high in* Salmonella typhi* and* Pseudomonas aeruginosa* while it was less in case of* E. coli*. Scintigraphic data also explained that 1 h after injection is the ideal time for scanning of infection (Figures 5, 6, and 7). The excretion of the radioactivity from organs is fast and there was no accumulation in any organs and after 24 hrs some of its quantity was observed at infection site and urinary bladder (Figure 7).

## 4. Conclusion

In present investigation, ^99m^Tc-ofloxacin freeze dried kits were developed via simple stirring of the ingredients at room temperature. Its freeze dried kits were highly stable, that is, for 24 h at room temperature and in blood serum at 37°C with long shelf life (>6 month) at 4°C and being safe to use with low colloidal amount. Biodistribution studies of the complex in infected rabbits showed hopeful results and it may be used in future in clinical tests for the diagnosis of bone, skin, sinus, lungs, airways, ear, and joint infections caused by the susceptible bacteria.

## Figures and Tables

**Table 1:** Effect of concentration of stannous chloride (reducing agent) and ofloxacin on the labeling efficiency (LE) of ^99m^Tc-ofloxacin (%LE ± SD, *n* = 3).

	Reducing agent					Ofloxacin				
	Amount (*μ*g)					Amount (mg)				
	25	50	75	100	125	0.5	1	1.5	2.0	2.5
Labelling efficiency (%)	80.23	95.50	99.20	97.7	95.17	84.75	95.5	99.7	97.1	96.90
Free ^99m^TcO_4_^−^	17.4	3.57	0.40	1.7	2.0	13.54	0.25	0.15	1.1	1.0
Colloids/hydrolyzed (^99m^TcO_2_)	2.37	0.93	0.40	0.6	2.8	1.30	0.25	0.16	1.8	2.1

**Table 2:** In vitro stability of ^99m^Tc-ofloxacin complex at room temperature and in blood serum at 37°C (%LE ± SD, *n* = 3).

	Room temperature						Serum at 37°C					
	Time (hrs)						Time (hrs)					
	1	2	3	4	5	6	1	2	3	4	5	6
Labelling efficiency (%)	99.62	99.60	99.18	99.09	99.01	98.81	99.07	99.03	99.02	99.01	99.00	99.00
Free ^99m^TcO_4_^−^	0.20	0.20	0.42	0.46	0.47	0.56	0.54	0.43	0.53	0.72	0.54	0.46
Colloids/hydrolysed (^99m^TcO_2_)	0.18	0.20	0.40	0.45	0.52	0.63	0.39	0.53	0.45	0.27	0.46	0.54

**Table 3:** Biodistribution of ^99m^Tc-ofloxacin in *E. coli* in rabbit (%ID/g ± SD, *n* = 3).

Organs	1 hr	2 hr	3 hr	4 hr
Thigh (target)	1.05 ± 0.22	1.01 ± 0.14	0.80 ± 0.15	0.65 ± 0.11
Thigh (NT)	0.44 ± 0.21	0.54 ± 0.11	0.43 ± 0.13	0.36 ± 0.15
Kidneys (RT)	3.68 ± 0.91	3.44 ± 0.89	2.37 ± 0.98	1.831 ± 0.87
Kidneys (LT)	4.04 ± 0.89	3.27 ± 0.82	4.01 ± 0.88	3.20 ± 0.86
Liver	2.96 ± 0.46	2.83 ± 0.31	0.81 ± 0.24	0.64 ± 0.20
Urinary bladder	58.69 ± 1.23	58.90 ± 1.21	66.11 ± 1.24	64.43 ± 1.40

**Table 4:** Biodistribution of ^99m^Tc-ofloxacin in *Pseudomonas aeruginosa* in rabbit (%ID/g ± SD, *n* = 3).

Organs	1 hr	2 hr	3 hr	4 hr
Thigh (target)	1.92 ± 0.22	1.31 ± 0.15	1.01 ± 0.10	1.03 ± 0.08
Thigh (NT)	0.52 ± 0.20	0.38 ± 0.14	0.31 ± 0.16	0.33 ± 0.11
Kidneys (RT)	2.76 ± 0.76	1.43 ± 0.72	1.33 ± 0.71	1.73 ± 0.76
Kidneys (LT)	2.12 ± 0.99	1.54 ± 0.89	1.50 ± 0.88	1.81 ± 0.78
Liver	2.86 ± 0.24	1.84 ± 0.25	1.43 ± 0.26	1.56 ± 0.22
Urinary bladder	34.76 ± 1.10	53.75 ± 1.02	38.17 ± 1.03	3.31 ± 1.02

**Table 5:** Biodistribution of ^99m^Tc-ofloxacin in *Salmonella typhi *(%ID/g ± SD, *n* = 3).

Organs	1 hr	2 hr	3 hr	4 hr	24 hr
Thigh (target)	1.80 ± 0.21	1.50 ± 0.15	1.31 ± 0.11	1.23 ± 0.12	0.39 ± 0.13
Thigh (NT)	0.44 ± 0.14	0.35 ± 0.07	0.31 ± 0.08	0.30 ± 0.09	0.15 ± 0.06
Kidneys (RT)	1.23 ± 0.91	1.69 ± 0.99	1.53 ± 1.10	1.51 ± 0.89	1.30 ± 0.88
Kidneys (LT)	1.16 ± 0.80	0.88 ± 0.88	1.02 ± 0.89	1.03 ± 0.88	1.78 ± 0.81
Liver	1.86 ± 0.22	1.67 ± 0.21	1.38 ± 0.25	1.28 ± 0.28	0.46 ± 0.27
Urinary bladder	64.99 ± 1.21	72.38 ± 1.20	72.31 ± 1.27	73.44 ± 1.28	3.12 ± 1.20
